# The Scaling and Modeling of Pay and the Robustness of the Effect of Core Self Evaluations on Career Success

**DOI:** 10.3389/fpsyg.2021.608858

**Published:** 2021-07-19

**Authors:** Yoav Ganzach, Asya Pazy

**Affiliations:** ^1^Ariel University, Ariel, Israel; ^2^Tel Aviv University, Tel Aviv, Israel

**Keywords:** pay, scaling, interaction models, constructive replications, logarithmic transformation

## Abstract

A number of recent studies used nominal pay in estimating the effects of individual differences, particularly core-self-evaluation, on career success. We show that this practice may lead to results that are substantively different from the results when the logarithm of pay is used. We conduct three constructive replications of previous studies, and argue that substantive conclusion based on the results of nominal pay are misleading.

## On the Scaling and Modeling of Pay: Three Constructive

The practice of using the logarithm of pay in estimating pay models is widespread. This is the case both in the management literature (e.g., 2009; Gerhart and Milkovich, 1989; Casey and Delquié, 1995; Judge et al., 1995, 1999; Stroh et al., 1996; Finkelstein and Boyd, 1998; Chatman et al., 1999; Kuhberger et al., 1999; Fulmer, 2009; Luhmann et al., 2011) and in the economic literature (in practically every paper published in the last 5 years in the *Journal of Labor Economics* – the most prominent journal in the area of remuneration research in economics – the logarithm of pay was used as the dependent variable). However, there is a growing number of papers in the management literature that deviate from this practice, and use nominal pay, rather than log pay, as a dependent variable. Recently this was particularly the case in studies that examined the effects of CSE (Core Self Evaluations) – a personality characteristic representing a combination of self-esteem, general self-efficacy, locus of control and neuroticism – on pay. Examples for such studies are: Judge and Hurst (2007,2008), Judge and Livingston (2008); Judge et al.(2009,2010); Resick et al. (2009), Grant and Wrzesniewski (2010); Stumpp et al. (2010), and Judge and Kammeyer-Mueller (2011). But using pay rather than log pay became more common also in studies that examined the effects of other individual differences such as the big-five personality dimensions (e.g., Sutin et al.,2009; Alfonsi et al., 2011; Spurk and Abele, 2011), as well as other individual differences such as self view (Hogue et al., 2010); self efficacy (Abele and Spurk, 2009); or social potency (Zhang and Arvey, 2009)^1^. In the current paper we demonstrate that this practice may lead to results that are considerably different from the results obtained by the traditional practice of applying a log transformation, and to different substantive conclusions. We examine what these differences are and when they occur.

### Why Log Pay?

There are a number of reasons for using log pay rather than nominal pay in pay models. First, the distribution of pay is skewed to the right, which violates the assumption of normality when estimating regression models. Second, quite often the variance explained in pay models is larger when a logarithmic rather than nominal pay scale is used. The third reason, a substantive reason, is more central to the current paper. It is based on the idea that the relationship between the construct (e.g., utility, satisfaction, or, particularly relevant to the current paper, career success) and its raw measure (i.e., nominal pay) exhibits a decreasing marginal sensitivity (see Hinrichs, 1969; Mitra et al., 2016, with regard to the relationship between pay and satisfaction. See Piliavin et al., 1986; Birenbaum, 1992, with regard to the relationship between pay and utility. See Judge et al., 1995; Seibert et al., 1999; Boudreau et al., 2001; Seibert et al., 2001 with regard to the relationship between pay and career success). Such a relationship is consistent with the idea that with regard to career success, percentage changes in pay, rather than nominal changes, matter to people, which was documented in a number of previous studies in the applied psychology literature (e.g., Zedeck and Smith, 1968; Worley et al., 1992; Mitra et al., 1997).

The idea that percentage change in pay, rather than nominal change, matters, call for a logarithmic pay scale. For example, a logarithmic pay scale suggests that the change associated with a pay increase from 10 to 20 is larger than the change associated with a pay increase from 100 to 110. On a logarithmic scale the former change is log(20)-log(10) = log(2)≈0.69, while the latter change is log(110)-log(100) = log(1.1)≈0.04. This difference between the two is not captured on a nominal pay scale, since on this scale both changes are equal to 10. Similarly, the intuition that the difference between two individuals, one earning 10 and the other 20, is larger than the difference between two other individuals, one earning 100 and one 110, is captured on a logarithmic pay scale, but not on a nominal pay scale.

The following citation from a recent paper by two Noble prize laureates provide a good summary of this idea (Kahneman and Deaton, 2010, p. 16489). “The logarithmic transformation represents a basic fact of perception known as Weber’s Law, which applies generally to quantitative dimensions of perception and judgment (e.g., the intensity of sounds and lights). The rule is that the effective stimulus for the detection and evaluation of changes or differences in such dimensions is the percentage change, not its absolute amount. In the context of income, a $100 raise does not have the same significance for a financial services executive as for an individual earning the minimum wage, but a doubling of their respective incomes might have a similar impact on both. The logarithmic transformation reveals an important regularity of judgment that risks being masked when a dollar scale is used.”

### Pay as an Indicator of Career Success

One possible view of pay as an indicator of career success is that the relationship between the two is linear, that is, that CS ≈ Pay, where CS is career success.

Another view is that the relationship between career success and pay, similar to other relationships between subjective perceptions and objective magnitudes, exhibits a decreasing marginal sensitivity; that is that changes in career success are related to *relative changes* in pay, or that ΔCS ≈ΔPay/Pay. By integrating both sides of this equation we obtain a logarithmic relationship between career success and pay, i.e., CS ≈ log(Pay).

The implications of this discussion is that the modeling of career success as a function of a vector of possible antecedents X should be based on equation 1 if pay is an indicator of career success and on equation 2 if log pay is an indicator of career success.

(1) $$\mathrm{Pay}=\alpha +\beta^{*}\,X$$

(2) log(Pay)=α+′βX*′

The decision whether to use equation 1 or equation 2 can be made based on statistical reasons (i.e., model fit, deviations from normality), or based on theoretical reasons (which relationship between career success and pay makes more theoretical sense). The purpose of the current paper is, however, more modest. We do not attempt to determine which model is ‘correct.’ Our purpose is to investigate the extent to which the results of pay models are robust to the pay scale by conducting constructive replications of data that were previously analyzed in the literature, and to examine which results are more sensitive and which are less sensitive to change in the pay scale. In our view, before changing the practice by which pay is modeled, the scale sensitivity of these models should be examined and inconsistencies between models based on the two types of scales should be reported.

#### A Note About the Terminology of Type I and Type II Errors in the Paper

For convenience we use in this paper a terminology implying that the true model is logarithmic. In this terminology Type I error implies detecting an effect in a nominal pay model when such an effect does not exist in the log pay model and type II error implies detecting an effect in log pay model when such an effect does not exist in a nominal pay model. Although by itself this terminology is neutral with regard to the question which is the more valid model, it obviously reflects our view that the log pay model is a more valid model. We emphasize, however, that the focus of the paper is not on the validity of the models that are analyzed, but on their robustness. We do, however, discuss questions regarding validity in the section “General Discussion.”

### Decreasing Marginal Sensitivity and Interactions vs. Main Effects in the Modeling of Pay

**FIGURE 1:**
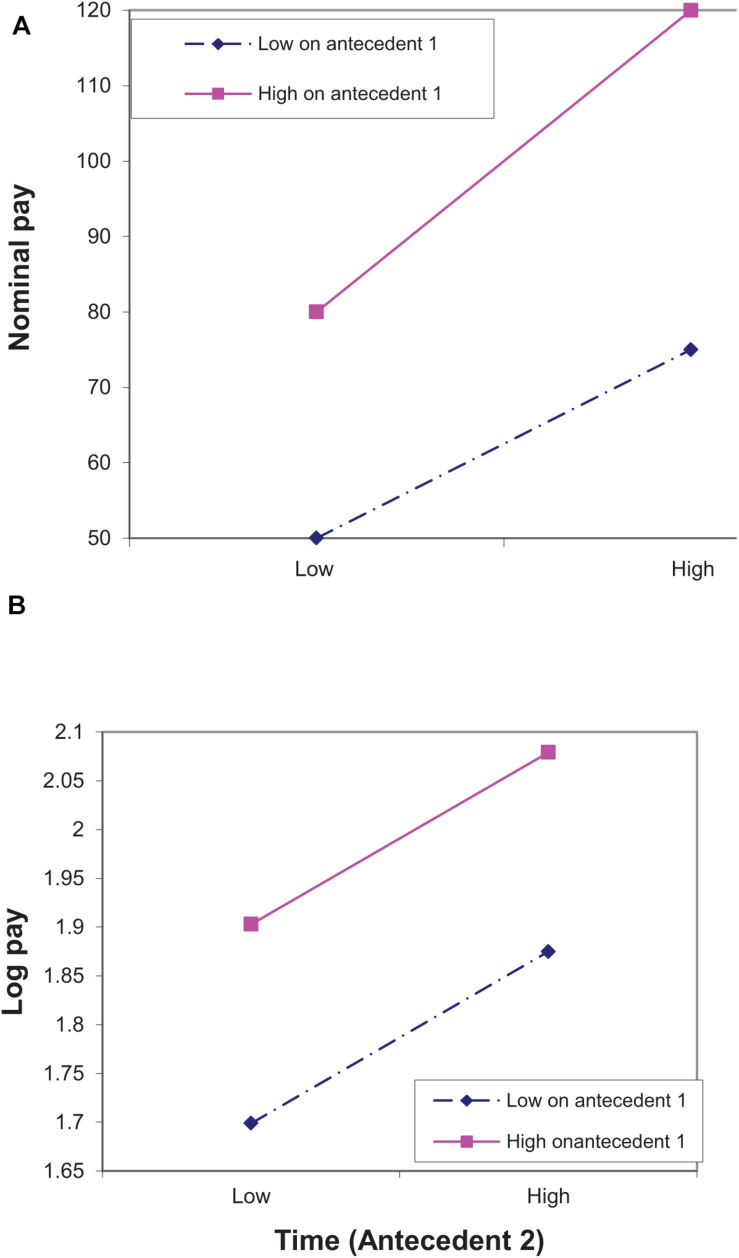
An illustration of Type I error in detecting interactions between two antecedents when the true model of pay is logarithmic. The antecedents can be either two individual characteristics or an individual characteristic and time. In **(A)** the dependent variable is nominal pay and in Figure 2A it is log pay. The points in **(B)** are associated with the points in **(A)**. Thus, for example, log(50) ≈ 1.7.

We turn now to an analysis of why decreasing marginal sensitivity leads to different results for a logarithmic pay scale than for a nominal pay scale, limiting our analysis to cases in which there is no cross-over interaction between antecedents in the determination of pay.^2^ Consider two individuals, one of them high and one of them low on a characteristic (e.g., sex) associated with pay, who had been paid 80 and 50, respectively, and gained an equal increase of 50% to 120 and 75. On a logarithmic pay scale, this pattern of pay increase does not indicate that the characteristic affects pay *growth*, since both advance by the same amount [i.e., log(75)-log(50) = log(120)-log(80) = log(1.5)]. On a nominal pay scale, however, the former advances more than the latter (40 vs. 25). Thus, if both time and the characteristic have a positive effect on pay growth, the characteristic × time interaction effect on nominal pay will be positive, even if there is no logarithmic interaction (i.e., no interaction on a logarithmic scale) between the two. In other words, if decreasing marginal sensitivity describes the relationship between pay and its antecedents, using nominal pay as a dependent variable may lead to a Type I error in detecting interactions. Figure 1 demonstrates this pattern by showing that on a nominal scale (Figure 1A) the pay growth of the individual high on the characteristic is steeper than the pay growth of the individual low on the characteristic, while on a logarithmic scale (Figure 1B) their pay growths are similar.

Our longitudinal example above referred to the interaction between time and a characteristic associated with pay (characteristic 1 in Figure 1). A similar argument is relevant to the interaction between two characteristics associated with pay in a cross sectional design. To see that, assume that Figure 1A,B represents four individuals, two of them, earning 120 and 75, are similar in that both are high on characteristic 1 (e.g., both are males) but are different in that the former is high and the latter is low on characteristic 2 (e.g., intelligence). The other two, earning 80 and 50, are both low on characteristic 1 (e.g., both are females), but high and low, respectively, on characteristic 2 (e.g., intelligence). In terms of log pay (percents), the difference within each pair is the same (60%), i.e., there is no characteristic × characteristic interaction. However in terms of nominal pay the difference in the first pair (45) is higher than the difference in the second pair (30), i.e., there is a characteristic × characteristic interaction.

**FIGURE 2:**
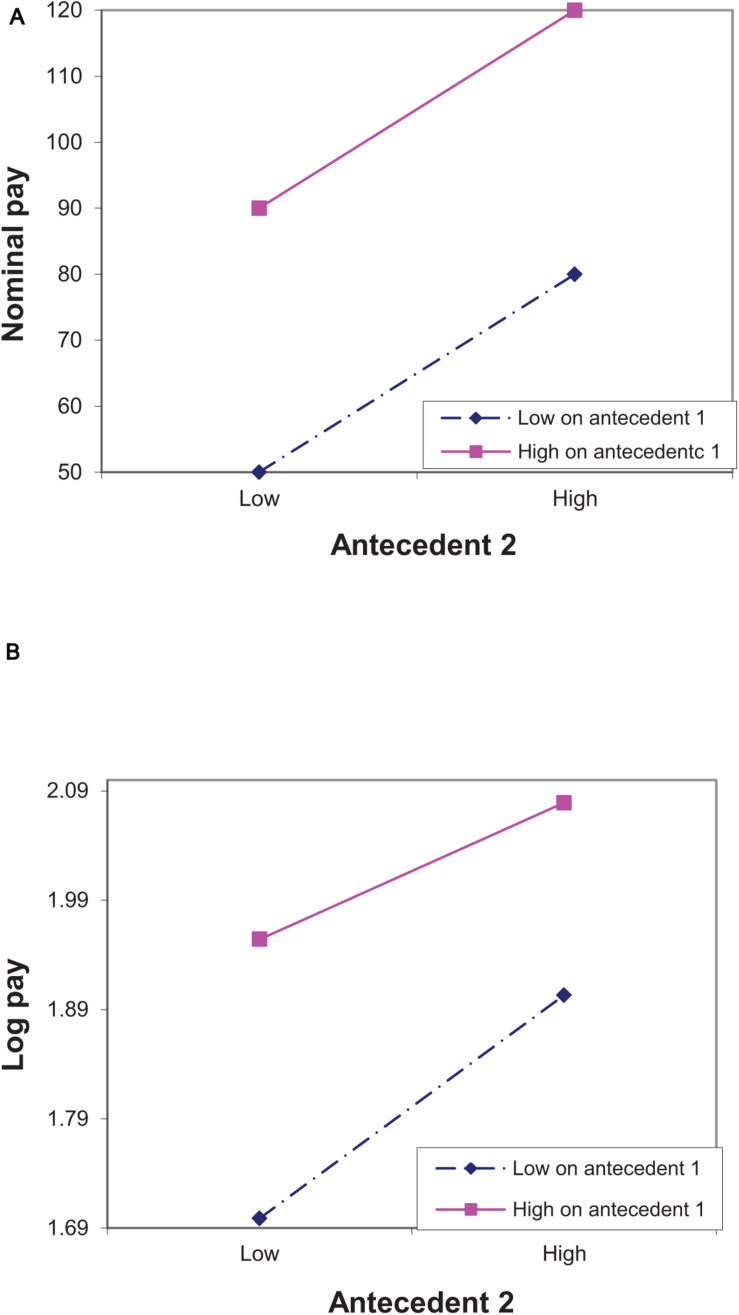
An illustration of Type II error in detecting interactions between two antecedents when the true model of pay is linear. The antecedents can be either two individual characteristics or an individual characteristic and time. In **(A)** the dependent variable is nominal pay and in **(B)** it is log pay. The points in **(B)** are associated with the points in **(A)**. Thus, for example, log(50) ≈ 1.7.

Figure 1 depicts a situation in which there is no logarithmic interaction and nominal pay models may exhibit spurious interaction. Another situation is that there is a logarithmic interaction, and using nominal pay eliminates this interaction. This situation is depicted in Figure 2A,B, in which there is an interaction on a logarithmic scale log(120)-log(90) < log(80)-log(50), but there is no interaction on a nominal scale (120-90 = 80-50). This is an example of a Type II error associated with using a nominal pay scale.

As our analysis focuses on cases in which there is no cross–over interaction, monotone transformations of the dependent variable in general, and logarithmic transformation in particular, do not considerably alter the main effects (see Dawes and Corrigan, 1974). This is evident both in Figures 1, 2 that show that changing the pay scale changed the pattern of interactions, but did not change the pattern of the main effects (although it may have changed the estimated effects). Thus, one feature of our analysis is that although using nominal pay is likely to lead to both Type I and Type II errors in detecting *interactions* between antecedents of pay, it is less likely to lead to such errors in detecting *main effects* of the antecedents.

A second feature of our analysis is that treating the pay scale as nominal, when it is really logarithmic, results in the effect of one antecedent on nominal pay appearing to be excessively stronger for those whose pay is higher as a result of being higher on another antecedent. This is similar to saying that if an antecedent affects percent differences, in terms of nominal pay, its effect will be more pronounced among highly paid than among lowly paid individuals. Indeed, the effect of antecedent 2 on nominal pay is stronger for those higher on antecedent 1 than for those lower on this antecedent, but its effect on log pay is similar for the two groups (Figure 1). Likewise, the effect of antecedent 2 on nominal pay is similar for those higher and those lower on antecedent 1, but its effect on log pay is *weaker* for those high on antecedent 1 (Figure 2).

These two features are particularly important since they suggest which previously published results are more likely, and which are less likely, to be influenced by the pay scale. First, they suggest that results involving main effects of nominal pay are more robust with regard to the pay scale in comparison to results involving interactions. And second they suggest that significant interactions of nominal pay models in which the influence of an antecedent on pay is stronger among those higher on another antecedent than among those lower on this antecedent is less robust to the pay scale than other types of interactions.

### Empirical Analysis

In the paper we examine the consequences of using nominal pay versus log pay in the context of constructive replications (Lykken, 1968) of three studies showing that the results of pay models are not robust to the pay scale. Two of these studies are replications are of previous studies that used nominal rather than logarithmic pay scale and examined the relationship between CSE and pay. The first (Judge and Hurst, 2008) tested in a cross-sectional design the hypothesis that CSE moderates the effect of parental socioeconomic status (pSES) and education on pay; that is, it examined the interaction between CSE and these two variables. The second (Judge and Hurst, 2007) tested in a longitudinal design the hypothesis that CSE moderates the effect of time on pay; that is it examined the interaction between CSE and time. We begin, however, with a constructive replication of previous studies about sex differences in return to education in which we examine the interaction between sex and education. Although this replication does not target specific papers in which the use of nominal- rather than log- pay may have led to erroneous conclusions, it allows us to compare interactions in nominal pay models to interactions in logarithmic pay models vis-à-vis the vast labor economics literature about sex-differences in return to education.

## Study 1

The literature on sex-differences in return to education in the US strongly suggests that females’ return to education is higher than males. Dougherty’s (2005) summary of the literature is that “Of the 27 studies, 18 report unambiguously higher schooling coefficients for females. Six report multiple estimates where the female coefficients are mostly higher. Two report mixed results that are evenly balanced. Only one reports higher schooling coefficients for males, and this study had a relatively small sample.” These findings in the US are also supported by findings in other countries (Trostel et al., 2002; Psacharopoulos and Patrinos, 2004), and are further supported by a number of theoretical explanations such as an inverse relationship between years of schooling and sex discrimination; a male–female differential in the quality of educational attainment; and occupational segregation of females into sectors where the returns to schooling are relatively high (Levy and Murnane, 1992).

Without exception, the studies reviewed above used logarithmic pay as a dependent variable, almost all of them used the log of hourly rate of pay. However, none compared the results of log pay models to the results of nominal pay models. In the current study we conduct such a comparison. We compare sex differences in the effect of education in log pay and nominal pay models.

### Method

#### Sample

The data were taken from the Wisconsin Longitudinal Study (WLS) of 10,317 randomly sampled Wisconsin students in the 1957 graduating high school class. Participants were surveyed in 1957, 1975, 1992, 2004, and 2011. The sample is broadly representative of males and females who had completed at least 12 years of education in Wisconsin. In the current analysis I use the 1992 wave of the survey when subjects were about 52 years old. The number of participants who completed the interview at this year was 8493.

#### Measures

##### Nominal pay

We used hourly rate of pay (in dollars) – the standard measure of pay in the labor economics literature– as our measure of pay.

##### Logarithmic pay

Logarithmic pay was the natural logarithm of nominal pay.

##### Educational attainment

We used the answer to a question “how many years of education do you have.”

##### Sex

Sex was coded as 1 for males and 2 for females.

##### Occupational prestige

The Duncan index was used as a measure for occupational prestige (Duncan, 1961).

### Results

Table 1 presents the means, standard deviations and inter-correlations of the study variable. It is clear from the table that the correlations of log pay with each of the determinants in this study (education, occupational prestige, and sex) was higher for log pay than for nominal pay, which is consistent with the argument that log pay should be preferred to pay as it supplies a better fit in pay models.

**TABLE 1:** Descriptive statistics and inter-correlations of the variables in Study 1.

	*n*	Mean	STD	1	2	3	4
(1) Nominal pay	7559	17.12	20.72	−			
(2) Log pay	7559	2.53	0.76	0.745	−		
(3) Sex	10317	1.52	0.50	–0.279	–0.427	−	
(4) Duncan index	8123	49.86	22.84	0.276	0.382	–0.069	−
(5) Education	8492	13.61	2.26	0.287	0.366	–0.159	0.490

The left side of Table 2 presents the results of two interaction models, one for log pay and one for nominal pay. In addition to sex, education and their interaction we introduced into the regression occupational prestige to control for occupational segregation between males and females that may have been quite substantial at this early cohort.

**TABLE 2:** Sex-differences in the return on education in nominal and logarithmic pay models in Study 1.

	Interaction models						Linear models					
	Nominal pay			Log pay			Nominal pay			Log pay		
Effect	Estimate	Standard error	*t*	Estimate	Standard error	*t*	Estimate	Standard error	*t*	Estimate	Standard error	*t*
Intercept	–17.10	4.093	4.2	2.483	0.134	18.5	4.222	1.586	2.7	2.167	0.052	41.8
Duncan index	0.160	0.011	14.5	0.009	0.0004	25.1	0.163	0.011	14.8	0.009	0.0004	24.9
Sex	4.982	2.706	1.8	–0.806	0.089	9.1	–10.102	0.441	2.9	–0.583	0.014	40.4
Education	3.023	0.298	10.1	0.034	0.010	3.5	1.457	0.111	13.2	0.058	0.004	15.9
Sex × Education	**−1.109**	**0.196**	**5.7**	**0.016**	**0.006**	**2.6**						

**t*-ratios above 2.6 are significant at the 0.01 level. *t*-ratios above 3.8 are significant at the 0.0001 level.*

It is clear from the table that with regard to the Sex × Education interaction, there are substantial differences between the log pay model and the nominal pay model. The interaction between education and sex is significantly *positive* in the log pay model (*p* < 0.01), and significantly negative in the *nominal pay* model (*p* < 0.0001). The positive interaction between sex and education in the log pay model implies that the return to education is higher among females than among males. The negative interaction between sex and education in the nominal pay model implies that the return to education is higher among males than among females.

The right hand side of Table 2 presents, respectively, the results of linear (main effects only) models of log pay and nominal pay. These two models are consistent with each other in that the relevant coefficients have the same sign and are significant in both models. Thus, whereas our analysis suggests that interactions in pay models are not robust with regard to the pay scale, it also suggests that main effects are rather robust. Note, however, that the *t*-values of the main effects in the linear model of log pay are larger than the corresponding *t*-values in the linear model of nominal pay. Since *t*-values are directly related to statistical power, this difference suggests that in smaller sample sizes even the robustness of main effects is not assured, and that log pay models have more statistical power than nominal pay models, and therefore are more robust even in main effects only models.

### Discussion

The results of the study demonstrate that interaction terms in pay models are not robust to the scaling of pay. In the current analyses the Sex × Education interaction was positive in the log pay model and negative in the nominal pay model. Two points are particularly worthwhile noting with regard to this difference. First, the interaction in the nominal pay model is the type of interaction that our analysis above suggests as more likely to be sensitive to what we called type I error, since it represents a case in which the influence of one antecedent (education) on pay is stronger among those higher on the other antecedent (sex – male) than those lower on this antecedent (sex –females). Second, while it is often not possible to determine what is the “true” sign of the interaction in pay models, this is not the case in the current study since previous theory and research suggests that return on education is higher among females, which implies (when females are coded as 2 and males as 1) a positive Sex × Education interaction. Thus, the negative sign of the interaction in the nominal pay model appears to be associated not only with a type I error as defined in the paper (detecting a lower effect of education on pay among females than males in the nominal pay model, when the effect in the log pay model is higher for females), but also with type I error as traditionally understood (detecting a lower effect of education on pay among females than males in the nominal pay model, when the *true* effect is higher for females).

## Studies 2 and 3: General Method

### Samples

The data for the studies reported here were all taken from the National Longitudinal Survey of Youth (NLSY), a national sample of Americans born between 1957 and 1964. We used this database since two of the studies we critique were based on this survey. The original sample of the NLSY included 12,686 participants. Due to funding constraints, 1,079 participants were dropped in 1984 and 1,643 in 1990. Natural sample attrition was about 10% a year. In Study 2, following Judge and Hurst (2007), we used observations from the five surveys between 1994 and 2002. In Study 3, following Judge and Hurst (2008), we used the 19 surveys between 1981 and 2004.

### Measures

#### Nominal Pay

We used hourly rate of pay (in cents) – the standard measure of pay in the labor economics literature– as our measure of pay.

#### Logarithmic pay

Logarithmic pay was the natural logarithm of nominal pay.

#### Educational Attainment

We used the answer to a question, asked in each of the surveyed years, about the highest grade ever completed.

#### Core Self Evaluations (CSE)

We used Judge and Hurst’s(2007;2008) measure of CSE, which was constructed from 12 items collected in the NLSY surveys. Two items, collected in the 1979 survey, were taken from Rotter’s (1966) internal–external locus of control measure. Five items, collected in the 1980 survey, were taken from Rosenberg’s (1961) self-esteem scale. Two items, collected in the 1987 survey, were taken from the Center for Epidemiological Studies Depression scale. Three items, collected in the 1992 survey, were taken from the Pearlin Personal Mastery Measure (Pearlin et al., 1981), which assesses the degree to which individuals perceive themselves in control of forces that impact their lives.

#### Self-Esteem

Rosenberg’s (1965) 10-items scale that was administered in the 1980 survey was used as a measure of self-esteem.

#### General Mental Ability (GMA)

The measure of GMA study was derived from participants’ test scores in the Armed Forces Qualifying Test (AFQT). This test was administered to groups of five to ten participants of the NLSY during the period of June through October 1980. Respondents were compensated, and the overall completion rate was 94%. The *GMA* score in the NLSY is the sum of the standardized scores of four tests: arithmetic reasoning, paragraph comprehension, word knowledge and mathematics knowledge, and is expressed as a percentile score from the general population.

#### Parental Socioeconomic Status (pSES)

Following Hauser(1994; see also Herrnstein and Murray, 1994; Bradley and Corwyn, 2002), our index for parental socioeconomic status includes four indicators: education of the two parents, parental family income, and occupational status of the parent holding the higher occupation. Parents’ education was measured in terms of the highest grade completed by each of the parents. Parental family income was based on the net family income in 1979 (it was excluded if the reported income for this year referred to the respondent’s own income). Parental occupational status was measured using the Duncan index which represents occupational prestige (Duncan, 1961). These four indicators were standardized and averaged to produce the narrow index of pSES.

#### Age, Gender Race, and Time

Age, sex, and race were collected at the first year of the survey. Ethnic background was coded as 0 if the participant was black or Hispanic, 1 if he or she was not. Time refers to the year in which the survey was conducted, the first year was coded as 0, the second as 1 and so on. Age refers to the age of the participant at 1979, the time of the first survey (note that this is a cross sectional age that does not change over time).

### Analyses

In each of the studies, we estimate the following type of models using a random coefficients modeling (we chose random, rather than fixed, modeling framework because by and large this was the framework that dominated the previous analyses we critique):

(3) Y=α+βX1+1βGMA2*+βX3+2γX1X1+2γGMA*2+c⁢o⁢n⁢t⁢r⁢o⁢l⁢s

where Y is either nominal pay or log pay, X_1_ is an individual characteristic that affects pay (sex in Study 1, CSE in Studies 2 and 3), GMA is General Mental Ability, and X_2_ is a moderator of the effects of both X_1_ and GMA on pay (education in Study 1, education and parental SES in Study 2, time in Study 3). The controls we use are, by and large, the standard controls in pay models within the context of each study. GMA plays a special role in our models because its importance in determining pay (e.g., Herrnstein and Murray, 1994) and the fact that it is highly correlated both with CSE, education and parental SES. Thus the inclusion of GMA in our models is necessary to rule out the possibility that interactions involving the focal antecedents we examine are not due to interactions involving GMA (Ganzach and Pazy, 2014; see also Judge et al., 2010, p. 101).^3^

We also note that, although to tried to adhere to the methods used in the original studies, in the spirit of constructive replication (e.g., Tsang and Kwan, 1999; Eden, 2002) we introduced some changes both in the measures and in the empirical design. These changes correct additional problems (i.e., problems unrelated to the use of log versus nominal pay) in the original studies (see Ganzach and Pazy, 2014 for these problems). However, for the central issue of the paper, these changes are unimportant, since studying the role of pay scale in pay models can be achieved by comparing the results of any reasonable nominal pay model to the results of an equivalent log pay model.

### Descriptive Statistics

**FIGURE 3:**
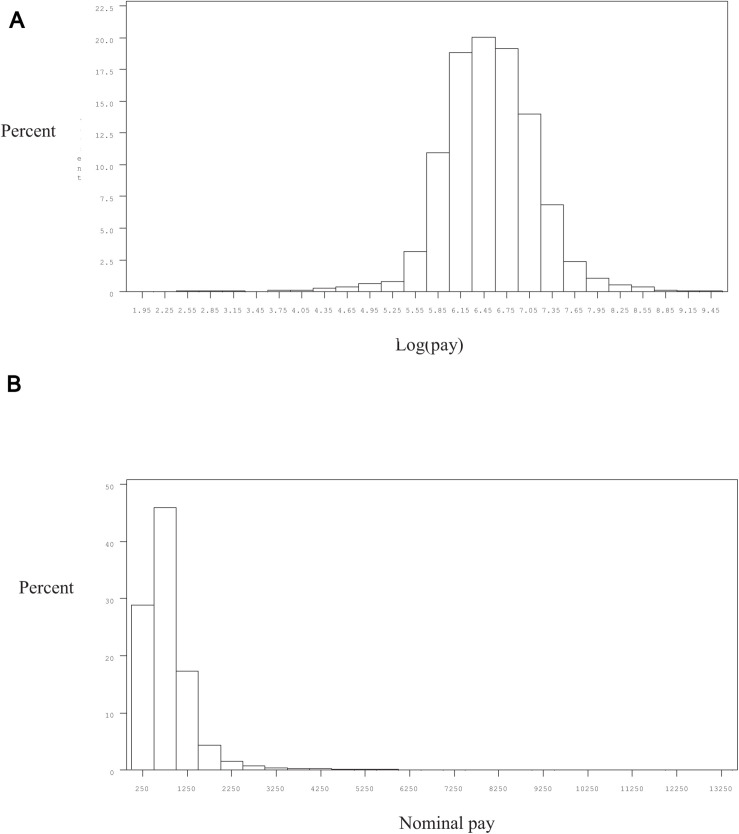
**(A)** The distribution of log pay in 1994. **(B)** The distribution of nominal pay in 1994.

Figure 3A presents the distribution of log pay and Figure 3B the distribution of pay in the 1994 survey (the pattern of the distributions in the other survey years was rather similar). It is clear from these figures that whereas the distribution of log pay is approximately normally distributed, the distribution of pay is skewed to the right. The skewness of the distribution of log pay is –0.28, whereas the distribution of pay is 5.72.

Table 3 presents descriptive statistics and inter-correlations among the variables in our studies. One aspect of the data that is apparent in this table is that the correlation of log pay with each of the determinants of pay (education, pSES, CSE, self-esteem, GMA, tenure, race, sex, and age) was higher for log pay than for nominal pay, which is consistent with the argument that log pay should be preferred to pay as it supplies a better fit in pay models. Note that in these correlations pay is essentially the dependent variable. However, there are also evidence that this is the case also when pay is the independent variable: The correlation of pay (the antecedent) and job satisfaction (the dependent variable) is higher for log pay than for nominal pay. (See Ganzach and Pazy, 2014, Table 1; see Greene, 1973, for the causal relationship between pay and job satisfaction and Dobrow et al., 2018 for a recent discussion).

**TABLE 3:** Descriptive statistics and inter-correlations of the variables in Studies 2 and 3.

	Mean	STD	1	2	3	4	5	6	7	8	9	10
(1) Nominal pay	730.3	541.7	−									
(2) Log pay	6.43	0.55	0.86	−								
(3) Education	12.6	2.3	0.34	0.38	−							
(4) pSES	−0.020	0.81	0.24	0.28	0.46	−						
(5) Sex	0.53	0.50	0.15	0.19	–0.09	–0.01	−					
(6) CSE	3.20	0.38	0.22	0.27	0.38	0.33	0.00	−				
(7) Self-esteem	3.30	0.40	0.17	0.21	0.31	0.25	–0.01	0.73	−			
(8) GMA	39.9	28.2	0.32	0.37	0.59	0.54	–0.02	0.46	0.35	−		
(9) Race	0.57	0.50	0.10	0.11	0.13	0.41	–0.01	0.14	0.08	0.45	0.15	−
(10) Age at 1979	19.6	2.20	0.05	0.07	0.06	0.05	–0.02	0.14	0.16	0.18	0.04	0.04

Another aspect of the data that is apparent in Table 3 is the high correlation between GMA and pay as well as the high correlations between GMA and the focal antecedents that are examined in the paper (education, pSES, CSE, and self-esteem), which highlight the need to control for GMA in assessing the effects of these characteristics on pay (see Judge et al., 2010; Ganzach and Pazy, 2014).

## Study 2

In this study, we examine the interaction between CSE and parental socioeconomic status (pSES) and the interaction between CSE and educational attainment in the determination of pay. These interactions were understood by Judge and Hurst (2007), who relied on a nominal pay model, as reflecting the ability of people with high CSE to ‘capitalize’ on advantages associated with pSES or educational attainment, reaping even more (in terms of pay) from such advantages than people with low CSE. As discussed above, when estimated from a nominal pay model, these interactions are susceptible to the fact that, to begin with, people with high educational attainment and high pSES are paid more than people low on these two variables.

### Results

We estimated both log pay and nominal pay models based on Judge and Hurst’s (2007) models, but, in the spirit of constructive replication, we introduced some changes which were, in our view, appropriate.^4^ As discussed above, as long as the nominal pay model and the log pay model are identical, these changes are not relevant to the study of the effect of pay scales on pay models. Similar to Judge and Hurst (2007), in these models advantage was conceptualized either as pSES or as educational attainment; that is, X_1_ in equation 3 is CSE and X_2_ is either pSES or education. Since the dependent variables (nominal pay and log pay) as well as one of our focal independent variables (education) are nested within subjects, we analyzed the data in an HLM framework in which these variables were treated as level 1 time varying variables, and sex, race, age at 1979, GMA CSE pSES were treated as level 2 variables.

The left side of Tables 4, 5 present, respectively, the results of interaction models of nominal pay and log pay when pSES or education are the independent variables interacting with CSE. There is no indication in the estimates of the log pay models that people with high CSE capitalize on advantages. Neither the interaction between CSE and education nor the interaction between CSE and pSES is significantly different from zero (*p* > 0.3, *p* > 0.5, respectively), suggesting that people with high CSE do not ‘capitalize’ either on their education or on their pSES. On the other hand, the interactions between CSE and education and CSE and pSES in the nominal pay model are positive (*p* < 0.01 for both), portraying a situation in which people with high CSE do capitalize on advantages. Note that these interactions are consistent with a pattern depicted in Figure 1 in which there is no interaction with regard to log pay but there is an interaction with regard to nominal pay, such that those who are high on one characteristic (those who are high on pSES or educational attainment) gain more from the other characteristic (CSE) than those who are low on this first characteristic (namely low on pSES or educational attainment).

**TABLE 4:** The interaction between CSE and education in nominal and logarithmic pay models in Study 2.

	Interaction models						Linear models					
	Nominal pay			Log pay			Nominal pay			Log pay		
Effect	Estimate	Standard error	*t*	Estimate	Standard error	t	Estimate	Standard error	*t*	Estimate	Standard error	*t*
Intercept	662.3	271.2	2.4	5.39	0.25	21.9	−531.3	65.6	8.1	4.99	0.064	77.8
Sex	198.3	9.3	21.2	0.242	0.009	25.8	201.7	9.38	21.5	0.243	0.009	26.0
Race	32.1	10.5	3.0	0.028	0.011	2.6	26.72	10.6	2.5	0.0265	0.0107	2.5
Age at 1979	−0.358	2.13	0.2	−0.0035	0.0021	1.6	0.270	2.14	0.1	−0.0033	0.0022	1.5
Education	−48.5	21.9	2.2	0.0266	0.0188	1.4	53.0	3.0	17.9	0.0600	0.0025	23.6
GMA	−6.88	1.24	5.6	0.0014	0.0011	1.3	4.31	0.25	16.9	0.00475	0.00024	19.3
CSE	−119.3	91.4	1.3	0.090	0.082	1.1	129.4	14.6	8.9	0.173	0.0146	11.9
Education*GMA	0.868	0.095	9.1	0.00025	0.00008	3.1						
Education*CSE	**21**.**7**	**7**.**4**	**2**.**9**	**0**.**0069**	**0**.**0063**	**1**.**1**						

**t*-ratios above 2.6 are significant at the 0.01 level. *t*-ratios above 3.8 are significant at the 0.0001 level.*

**TABLE 5:** The interaction between CSE and parental socioeconomic status (pSES) in nominal and logarithmic pay models in Study 2.

	Interaction models						Linear models					
	Nominal pay			Log pay			Nominal pay			Log pay		
Effect	Estimate	Standard error	*t*	Estimate	Standard error	*t*	Estimate	Standard error	*t*	Estimate	Standard error	*t*
Intercept	−34.8	71.0	0.5	5.70	0.061	93.1	49.8	71.3	0.7	5.72	0.06	93.8
Sex	203.1	11.2	18.1	0.223	0.011	22.6	205.5	11.4	18.1	0.220	0.010	22.7
Race	−60.2	13.3	4.5	−0.0543	0.0115	4.7	−70.5	13.4	−5.2	−0.0573	0.011	5.0
Age at 1979	−4.78	2.59	1.9	−0.0082	0.0022	−3.7	−4.11	2.61	−1.6	−0.00801	0.00223	3.6
pSES	−193.1	62.8	3.1	0.0202	0.0541	0.4	98.2	8.8	11.2	0.0840	0.0075	11.2
GMA	6.60	0.28	23.8	0.00689	0.00023	28.8	7.30	0.27	26.9	0.00711	0.00023	30.6
CSE	201.9	17.7	11.4	0.214	0.015	14.0	176.3	17.8	9.9	0.207	0.015	13.6
pSES*GMA	2.59	0.29	8.9	0.00083	0.00025	3.3						
pSES*CSE	**60.0**	**21.0**	**2.9**	**0.011**	**0.02**	**0.6**						

**t*-ratios above 2.6 are significant at the 0.01 level. *t*-ratios above 3.8 are significant at the 0.0001 level.*

The right sides of Tables 4, 5 present, respectively, the results of linear (main effects only) nominal and log pay models. The linear models of nominal pay are consistent with the linear models of log pay in that the coefficients have the same sign and are significant. Thus, as in Study 1, in this study too, the main effects are robust with regard to the pay scale, the interactions are not. However, note that the *t*-values of the main effects in the linear model of log pay are larger than the corresponding *t*-values in the linear model of nominal pay, suggesting that in smaller sample sizes, type-I errors are more frequent when nominal rather than log pay is used, even in the detection of main effects^5^.

### Discussion

Our results suggest that the findings about the interaction between pSES and CSE and the interaction between educational attainment and CSE (Judge and Hurst, 2007) are not robust to the pay scale used. These interactions are significant when a nominal pay scale is used and non-significant when a logarithmic scale is used. Thus, in these data, interactions are not robust to the pay scale.

Similar to Study 1, our findings in this study are consistent with the pattern in which the effect of an antecedent (CSE) appears to be stronger for the higher paid group in the nominal pay model than in the log pay model. The effect of CSE is stronger for people coming from higher pSES background and who have more education, i.e., those who populate the higher paid groups.

In this context we note that although interactive models are often appealing to researchers, as they represent interesting theories about the relationship between antecedents and outcomes, linear models usually provide a more adequate description of actual relationships between variables (e.g., Meehl, 1959; Goldberg, 1970; Dawes and Corrigan, 1974; Ganzach, 1994, 1997a). Furthermore, we note that other things being equal, linear models should be preferred on interactive models on the basis of parsimony, the principle that a superior theory is this which achieves maximum explanatory and predictive value while invoking a minimum number of entities and relationships (e.g., Mulaik et al., 1989; Cheung and Rensvold, 2001).

## Study 3

Judge and Hurst (2008) found that in a nominal pay model, the Time × CSE interaction is significantly positive. Since such interaction implies that the pay of individuals with higher CSE grows faster over time than the pay of individuals with lower CSE, they interpreted this result as suggesting that the higher the CSE, the stronger the growth in career success. In the current study we conduct a constructive replication of this study. In this replication we compare the interaction between *self esteem* – the major component of CSE (Judge et al., 2003) – and time in a nominal pay model to this interaction in a log pay model^6^. Again, we adhered to the methods used in the original study we critique, but introduced some changes both in the measures and in the empirical design^7^. Some of the changes are based on Ganzach and Pazy (2014) literal replication of Judge and Hurst (2008)^8^, while other are relatively minor changes that are aimed to improve on the original methods. However, as mentioned above, for studying the role of pay scale in pay models, these changes are unimportant.

### Results

The left side of Table 6 presents the results of two interaction models, one for log pay and one for nominal pay. The models are based on Equation 3 in which X_1_ is CSE and X_2_ is time (survey year). The data were analyzed within Hierarchical Linear Modeling framework (Bryk and Raudenbush, 1987) in which pay, time and education were treated as level 1 variables. Sex, race, GMA, CSE, pSES and Age at 1979 were treated as level 2 variables.

**TABLE 6:** The interaction between self-esteem and time in nominal and logarithmic pay models in Study 3.

	Interaction models						Linear models					
	Nominal pay			Log pay			Nominal pay			Log pay		
Effect	Estimate	Standard error	*t*	Estimate	Standard error	*t*	Estimate	Standard error	*t*	Estimate	Standard error	*t*
Intercept	−131.9	34.2	3.9	5.22	0.04	122.9	−309.1	28.4	10.9	5.10	0.03	137.2
Sex	130.9	4.8	27.3	0.211	0.006	33.3	132.9	4.8	27.6	0.213	0.006	33.5
Race	−20.57	5.45	3.8	−0.038	0.007	5.2	−20.38	5.47	−3.7	−0.036	0.007	5.0
Age at 1979	18.04	1.11	16.2	0.016	0.001	10.9	17.40	1.12	15.5	0.015	0.001	10.3
GMA	0.740	0.134	5.5	0.0040	0.0001	24.4	3.46	0.10	33.9	0.0060	0.0001	45.4
Time	−6.27	3.07	2.0	0.010	0.003	3.9	22.89	0.38	59.3	0.025	0.0003	80.4
Self-esteem (SE)	47.94	8.91	5.4	0.104	0.011	9.6	−309.1	28.4	10.9	0.116	0.009	13.6
Time*GMA	0.426	0.013	31.2	0.00026	0.00001	22.5						
Time*SE	**3.67**	**0.97**	**3.8**	**0.0015**	**0.0008**	**1.9**						

**t*-ratios above 2.6 are significant at the 0.01 level. *t*-ratios above 3.8 are significant at the 0.0001 level.*

It is clear from the results in Table 6 that whereas the interaction between self-esteem and time is significant in the nominal pay model (*p* < 0.0001), it is not significant in the log pay model (*p* > 0.4). In addition, similar to the first two studies, the coefficients of the linear model of nominal pay are consistent with the coefficients of the linear models of log pay (see the right side of Table 6). However, and in agreement with the first two studies, the *t*-values of the main effects in the linear model of log pay are larger than the corresponding *t*-values in the linear model of nominal pay.

### Discussion

Our results suggest that the finding about the interaction between time and CSE are not robust to the pay scale used. They reveal an interactive relationship when nominal pay is used and an additive relationship when a logarithmic pay is used. Again, the results of the main effects models show that whereas pay models are sensitive to the pay scale with regard to interaction effects, they are less sensitive to the scale with regard to main effects.

As in Studies 1 and 2, our findings are consistent with the pattern in which the effect of an antecedent (time) appears to be stronger for the higher paid group in the nominal pay model than in the log pay model. Indeed, the effect of time is stronger for people with higher self-esteem, who enjoy higher pay already at the beginning of their career (Indeed, the results presented in Table 6 which suggests that self-esteem has a positive effect on the intercept of time, an effect that is robust to the pay scale used. Since time was coded as 0 for the first survey year, this effect indicates a positive effect of self-esteem on initial pay).

Finally, Table 6 suggests that whereas the Time × Self-esteem interaction is not significant in the log pay model, the Time × GMA interaction is significantly positive. Thus, the conclusion of Judge et al. (2010) that there is a positive relationship between GMA and growth in career success, which was based on using nominal pay as a dependent variable, is – unlike the conclusion about positive relationship between CSE and growth in career success (Judge and Hurst, 2008) – robust to the pay scale that is used. Note however that the effect size of the Time × GMA interaction is substantially reduced in the log pay model in comparison to the pay model (*t*-values of 22.5 and 31.2, respectively), suggesting that part of the influence of GMA on growth in success estimated by Judge et al. (2010) is due to the pay scale they used.

## General Discussion

The results reported in this paper suggest that it is desirable that in testing hypotheses regarding pay on the basis of nominal pay, particularly interaction hypotheses, robustness checks using log transformation of pay will be conducted. Furthermore, it is our view that since log transformation is the common practice in research involving pay, strong arguments for adopting the unconventional practice of using nominal pay scale should be put forward.

It is rather surprising, therefore, that quite a few papers in top journals adopted the use of nominal pay without carefully looking into the robustness of this practice. Our attempt to discover how this practice creeped into the literature led us to the original paper that seemed established it (Judge and Hurst, 2007). In their paper, Judge and Hurst did not conduct a robustness check of this practice, but rather suggested that there are two papers – Busemeyer and Jones (1983) and Russell and Dean (2000) – that justify the use of nominal- rather than log- pay (see Judge and Hurst, 2007, p. 1216). However, it seems to us that Judge and Hurst misunderstood the implications of these two papers to the scaling of pay in pay models. First, Busemeyer and Jones (1983) suggest that for interactions in multiple regressions to be meaningful (i.e., reflect the interactive relationships between the constructs), the functional relationship between the measurement of the dependent variable and the underlying construct should be linear. If one suspects that this relationship is not linear, an appropriate transformation is necessary. Thus, if anything, Busemeyer and Jones (1983) are in favor of using transformations when measurement theory calls for it. In the other paper that discussed the use of nominal pay, Russell and Dean (2000) do indeed argue, as Judge and Hurst(2007, p. 1216) suggest, that “log transformations of positively skewed dependent variables ‘greatly enhance’ the probability of committing a Type II error.” However, Russell and Dean (2000) analyze a situation in which the relationship between the underlying construct and the measurement (of the dependent variable) is linear. It is, however, not at all relevant to a non-linear relationship. If such a relationship exists, *not* transforming the measurement will increase, rather than decrease, the probability of type II error. Interestingly enough, even when arguing against logarithmic transformation in general, Russel and Dean also think that pay is an exception. In this very same paper they state that they are “…unaware of any studies … providing a theoretical rationale justifying non-linear (monotonic or non-monotonic) transformations in applied psychological or management research *(although concepts like the diminishing marginal utility of money may provide such a rationale in the future)*.” (p. 168; our italics).

The need for log transformation of pay, or at least for robustness checks involving a transformation when nominal pay is used as a dependent variable, is not always obvious, since in many occasions the results of nominal pay models are not very different from the results of log pay models. Yet, in some circumstances they are considerably different. This is particularly true when interactions are involved. However, log transformation is also desirable in models involving only main effects. First, as our results show, models in which log pay is the dependent variable have better fit than models in which nominal pay is the dependent variable. Second, as our results also show, nominal pay, but not log pay, is strongly skewed, which suggests violation of the regression assumptions.

From a substantive point of view, log transformation is appropriate for pay since relative, rather than absolute, change in pay is important to people (Zedeck and Smith, 1968; Worley et al., 1992; Mitra et al., 1997). Under this assumption, a logarithmic transformation creates an equal interval scale in which similar differences are of the same magnitude (Thurstone, 1929; Stevens, 1946). This idea could also be understood in terms of the distinction between the underlying concept (career success) and its measurement (pay). Career success cannot be equated with nominal pay, and is better understood as a perceptual variable associated with the *perception* of pay. Therefore, a logarithmic function seems to be appropriate to describe the relationship between career success and nominal pay in the same way that the logarithmic function is commonly used to describe the relationship between perceptions (e.g., utility, loudness) and the physical characteristics associated with them (e.g., money, sound intensity). In this context one can think about psychophysical methods as a way by which the functional relationship between career success and pay as its measure could be validated. For example, subjects may rate apparent distance between two or more rates of pay and by that partitioning the pay continuum into apparently equal intervals; or they may directly judge the magnitude or level of career success based on rate of pay (Stevens, 1958).

It is interesting to compare our treatment of career success as a perceptual variable to the treatment of well-being as a perceptual variable. Essentially what Kahneman and Deaton (2010) argue when recommending log transformation of income in modeling the relationship between income and well-being (as discussed in the introduction) is that what affect the way we appraise our well-being is not income itself, but rather the perception of income, which is best estimated as logarithmic function of income. Our argument is similar. We argue that what affect our appraisal of career success is not pay itself, but our perception of pay, which is best estimated as a logarithmic function of pay.

We note, however, that there may be occasions in which modeling nominal pay may be of interest. Thus, when pay raises are given in percentage it is indeed the case that the ‘rich get richer’ (Judge and Hurst, 2008). This may be of concern, for example, for those who represent the less paid workers in labor negotiations in which percentage increases are considered. However, if one is interested in the psychological or sociological processes underlying career success, modeling nominal pay is of less interest since most often such models simply reflect mundane labor market practices of granting pay increases, or determining differential wages, in percentage terms^9^ – practices stemming from the fact that relative changes are what people care about. Thus, that ‘the rich get richer’ does not require any substantive theory. It is most likely due to the fact that the salaries of those whose pay is higher grow more in terms of nominal pay when pay raises are granted in percentages. Similarly, GMA has a stronger effect on nominal pay among males than among females because GMA-related differential pay is determined in percentage terms, and males have a higher pay level to begin with. Likewise, CSE is likely to show a stronger effect on nominal pay for those who come from high pSES or are more educated, not necessarily because they are better in capitalizing on their pSES or education, but simply because they enjoy a higher base pay than the latter. On the other hand, findings that are based on interactions obtained from logarithmic pay models may be associated with non-trivial labor market process having theoretical implications. Thus, the interaction between GMA and time in log pay models may reflect ability induced gravitational processes in the labor market (Wilk et al., 1995; Wilk and Sackett, 1996), or the interaction between sex and education may reflect processes of sex discrimination or sex based occupational segregation (e.g., Levy and Murnane, 1992).

The results presented in the current paper suggest a need for a re-examination of recent studies that used nominal pay as dependent variable. As our analysis indicates, the results of studies involving interactions (e.g., Judge and Hurst, 2007, 2008; Judge and Livingston, 2008; Abele and Spurk, 2009; Judge et al., 2010) are most susceptible to Type I errors. Results of models that examined only linear relationships between pay and its antecedents (e.g., Judge et al., 2009; Zhang and Arvey, 2009) are less likely to suffer from such errors, although the size of the relevant effects may be biased.

The pattern of interaction most likely to be associated with Type I errors in nominal pay models is an interaction in which the pay of those who are high on one antecedent is influenced more by the other antecedent than the pay of those who are low on the other antecedent (Figure 1A,B). Of the five recent studies that examined interactions using nominal pay we are aware of, the two studies that were examined in Studies 2 and 3 here (Judge and Hurst, 2007, 2008) display this pattern. But the other three also conform to this pattern. Thus, the interaction in Judge et al. (2010) is associated with higher paid people (more intelligent as opposed to less intelligent) advancing more in their pay over time, the interaction in Abele and Spurk (2009) is associated with higher paid people (those higher on self efficacy as opposed to those low on self-efficacy) advancing more in their pay over time; and the interaction in Judge and Livingston (2008) is associated with the pay of higher paid people (males as opposed to females) being influenced more by family attitudes.

Finally, the discussion of the merits of using logarithmic pay scale vs. nominal pay scale presented in this paper is relevant to other measures in which percent change, rather than absolute change, is meaningful (see Mosteller and Tukey, 1977, p. 91). For example, a log transformation may be appropriate for variables such as reaction time (Fishbach et al., 2003; Robinson and Tamir, 2005); number of symptoms (Loehlin et al., 2003); illness duration (Holliday et al., 2006); number of mating partners (Jonason et al., 2009); firm size (see Van Dyck et al., 2005, for using a logarithmic transformation; Turban and Greening, 1996, for not using such a transformation) or the number of contacts made by sales-people (see Brown and Peterson, 1994, for using a logarithmic transformation; Grant and Wrzesniewski, 2010 for not using such a transformation) to name just a few examples. Thus, in contrast to Carte and Russell (2003) who argued that the non-linear (logarithmic) transformation of pay is “The only exception [to logarithmic transformation] we are familiar with is the notion of marginal decreasing utility of money from labor economics” (p. 491), we believe that there are a number of situations in which logarithmic (or another non-linear) transformation should be considered. This view is consistent with a number of studies that suggest that non-linear transformations in general, and logarithmic transformation in particular, are often justified by substantive theory (e.g., Busemeyer and Jones, 1983; Jaccard et al., 1990; Lubinski and Humphreys, 1990; McClelland and Judd, 1993; Ganzach, 1998).

## Data Availability Statement

The original contributions presented in the study are included in the article/supplementary material, further inquiries can be directed to the corresponding author/s.

## Ethics Statement

The studies involving human participants were reviewed and approved by Moshe Leshno Tel Aviv University. The patients/participants provided their written informed consent to participate in this study.

## Author Contributions

YG and AP designed the studies, analyzed the data, and wrote the manuscript. Both authors contributed to the article and approved the submitted version.

## Conflict of Interest

The authors declare that the research was conducted in the absence of any commercial or financial relationships that could be construed as a potential conflict of interest.

## Footnotes

1 Included are also studies in which participants rated their pay on a numerical scale which was linearly related to their nominal pay (e.g., a pay between 0 and 10 rated as 1, between 10 and 20 as 2 and so on. See for example Judge et al., 2009). With regard to the issues discussed in the paper, these studies are not different from studies that use nominal pay. Note also that there are also earlier studies that used nominal pay. See, for example Parhhiban et al. (1998) and Gomez-Meijia et al. (2003). Our impression is, however, that this practice was rarer in previous years.

2 In most contexts of pay research the relationships between pay and its antecedents do not display cross over interactions since higher values on the antecedent predict higher values of pay independently of the values of other variables (although the strength of this relationship may depend on the values of other variables leading to ordinal interaction). Thus, for example, in modeling pay a ‘positive’ individual characteristic such as General Mental Ability or emotional stability is considered to be universally associated with pay.

3 As additional controls, quadratic terms can be introduced into the model to account for non-linearity in the measurement of in the independent variable, or for non-linearity in the relationship between the independent variables and the dependent variables (Lubinski and Humphreys, 1990; Ganzach, 1997b; see Cohen et al., 2013, pp. 299–300 for a review). For the sake of consistency with the previous literature which by and large did not include quadratic terms, and to keep the presentation simple, we did not include quadratic terms of the independent variables in our models. However, unless otherwise reported, the conclusions from our analyses are not changed by introducing these terms.

4 The main difference between our analysis and Judge and Hurst’s (2007) is that we control for GMA and the interaction between GMA and the advantage variables, while they did not. Other differences are: (1) We apply a time-varying multi-level model whereas Judge and Hurst averaged the variables over the five 1994–2002 surveys and use a single-level model. A time-varying model is, in our view, more appropriate for the data. (2) We used hourly rate of pay rather than total net family income, and therefore did not control for spouse’s income and number of hours worked (3) Judge and Hurst (2007) use a composite of years of education, SAT score and high school GPA as a measure of educational attainment, whereas we used the simpler measure of years of education. We prefer this latter operationalization because (a) This is the common operationalization of educational attainment in the literature; (b) SAT and high school GPA are missing for about 90% of the subjects, which not only creates a problem of sampling bias, but considerably reduces the number of subjects available for the analysis; and (c) Judge and Hurst’s (2007) operationalization, but not ours, implies that educational attainment is constant over time, which does not allow for time-varying modeling of the relationship between education and pay. (4) Our measure of pSES is somewhat different than Judge and Hurst’s (2007) as this is the more accepted measure in pay research (Herrnstein and Murray, 1994; Bradley and Corwyn, 2002); and (5) we controlled for cross-sectional age whereas Judge and Hurst (2007) did not.

5 Tables 4, 5 also suggest that whereas the Education × CSE and pSES × CSE interactions are not significant, the Education × GMA and pSES × GMA interactions are significant. This may suggest that whereas people with high CSE do not capitalize on advantages, people with high GMA do. However, to establish the validity of such interactions when the components of the interaction are correlated (see Table 3 for the correlations between GMA and education and pSES), it is necessary to control for the quadratic terms of the components (see footnote 3). When these quadratic terms are introduced, the Education × GMA and pSES × GMA interactions are not significant, suggesting that the interactions detected in a reduced model that does not include quadratic terms are spurious.

6 We chose to use self-esteem rather than CSE because the inappropriate temporal order between the Judge and Hurst (2007, 2008) measurement of CSE and the measurement of pay, as 7 out of the 12 items of their CSE measure were taken during or after the measurement of pay (see Ganzach and Pazy, 2014).

7 The major changes were: (1) The introduction of GMA and the GMA × time interaction as a control; (2) Controlling the temporal order between the independent and dependent variables (by using self-esteem, which was measured prior to the measurements of pay, rather than CSE). The minor changes were (1) Omitting from the analysis subjects who are enrolled in school. Not omitting them may inflate the mobility of individuals high on an antecedent (e.g., high GMA or self-esteem), since such individuals are likely to hold less favorable temporary jobs at earlier ages while being enrolled in school, resulting in inflated slopes associated with leaving school and taking ‘regular’ jobs rather than real shifts in career success; and (3) Using the 1981–2004 surveys (Judge and Hurst, 2007 used the 1979–2004 surveys), to ensure the temporal order between self-esteem (that was measured in 1980) and the measurement of pay.

8 Ganzach and Pazy (2014) replication showed that the Time × CSE interaction disappears and even reversed when (1) Intelligence is controlled for, (2) the temporal order between CSE and pay is maintained, and (3) the logarithm of pay is used as dependent variable. However, Ganzach and Pazy (2014) did not directly compare the consequences of using pay rather than log pay.

9 Note also that if pay raises are granted in percents, the distribution of pay raises is likely to be approximately normal, which leads to a skewed cross-sectional distribution of nominal pay. Viewed from this perspective, the log transformation can be understood as necessary to capture the process underlying the distribution of nominal pay.

## References

[B1] Abele A. E. Spurk D. (2009). How do objective and subjective career success interrelate over time? *J. Occup. Organ. Psychol.* 82 803–824. 10.1348/096317909x470924

[B2] Alfonsi G. Conway M. Pushkar D. (2011). The lower subjective social status of neurotic individuals: multiple pathways through occupational prestige, income, and illness. *J. Pers.* 79 619–642. 10.1111/j.1467-6494.2011.00684.x 21534966

[B3] Birenbaum M. H. (1992). Issues in utility measurement. *Organ. Behav. Hum. Decis. Proc.* 52 319–330. 10.1016/0749-5978(92)90024-2

[B4] Boudreau J. W. Boswell W. R. Judge T. A. (2001). Effects of personality on executive career success in the United States and Europe. *J. Voc. Behav.* 58 53–81. 10.1006/jvbe.2000.1755

[B5] Bradley R. H. Corwyn R. F. (2002). Socioeconomic status and child development. *Annu. Rev. Psychol.* 53 371–399. 11752490 10.1146/annurev.psych.53.100901.135233

[B6] Brown S. P. Peterson R. A. (1994). The effect of effort on sales performance and job satisfaction. *J. Mark.* 58 70–80. 10.2307/1252270

[B7] Bryk A. S. Raudenbush S. W. (1987). Application of hierarchical linear models toassessing change. *Psychol. Bull.* 101 147–158. 10.1037/0033-2909.101.1.147

[B8] Busemeyer J. R. Jones L. E. (1983). Analysis of multiplicative combinationrules when the causal variables are measured with error. *Psychol. Bull.* 93 549–562. 10.1037/0033-2909.93.3.549

[B9] Carte T. A. Russell C. G. (2003). In pursuit of moderation: nine common errors and their solutions. *MIS Q.* 27 479–501. 10.2307/30036541

[B10] Casey J. T. Delquié P. (1995). Stated vs Implicit Willingness to Pay Under Risk. *Organ. Behav. Hum. Decis. Process.* 61 123–137. 10.1006/obhd.1995.1010

[B11] Chatman J. A. Caldwell D. F. O’Reilly C. A. (1999). Managerial personality and performance: a semi-idiographic approach. *J. Res. Pers.* 33 514–545. 10.1006/jrpe.1999.2263

[B12] Cheung G. W. Rensvold R. B. (2001). The effects of model parsimony and sampling error on the fit of structural equation models. *Organ. Res. Methods* 4 236–264. 10.1177/109442810143004

[B13] Cohen J. Cohen P. West S. G. Aiken L. S. (2013). *Applied Multiple Regression/Correlation Analysis for the Behavioral Sciences.* London: Routledge.

[B14] Dawes R. M. Corrigan B. (1974). Linear models in decision making. *Psychol. Bull.* 81 95–106.

[B15] Dobrow R. S. Ganzach Y. Liu Y. (2018). Time and job satisfaction: a longitudinal study of the differential roles of age and tenure. *J. Manag.* 44 2558–2579. 10.1177/0149206315624962

[B16] Dougherty C. (2005). Why are the returns to schooling higher for women than for men? *J. Hum. Resour.* 4 969–988. 10.3368/jhr.xl.4.969

[B17] Duncan O. D. (1961). “A socioeconomic index for all occupations,” in *Class: Critical Concepts*, Vol. 1 ed. Scott J. (Milton Park: Taylor and Francis), 388–426.

[B18] Eden D. (2002). Replication, meta-analysis, scientific progress, and AMJ’spublication policy. *Acad. Manag. J.* 5 841–846.

[B19] Finkelstein S. Boyd B. K. (1998). How much does the CEO matter? The role of managerial discretion in the setting of CEO compensation. *Acad. Manag. J.* 41 179–199. 10.2307/257101

[B20] Fishbach A. Friedman R. S. Kruglanski A. W. (2003). Leading us not into temptation: momentary allurements elicit overriding goal activation. *J. Pers. Soc. Psychol.* 84:296. 10.1037/0022-3514.84.2.296 12585805

[B21] Fulmer I. S. (2009). The elephant in the room: labor market influences on CEO compensation. *Pers. Psychol.* 62 659–695. 10.1111/j.1744-6570.2009.01154.x

[B22] Ganzach Y. (1994). Theory and configurality in expert and layperson judgment. *J. Appl. Psychol.* 79 439–448. 10.1037/0021-9010.79.3.439 9638091

[B23] Ganzach Y. (1997a). Configurality in judgment: is it a bias. *Psychonom. Bull. Rev.* 4 382–386. 10.3758/bf03210797

[B24] Ganzach Y. (1997b). Misleading interaction and curvilinear terms. *Psychol. Methods* 3 235–247. 10.1037/1082-989x.2.3.235

[B25] Ganzach Y. (1998). Nonlinear models in decision making: the diagnosis of psychosis versus neurosis from the MMPI. *Organ. Behav. Hum. Decis. Process.* 74 53–61. 10.1006/obhd.1998.2752 9705812

[B26] Ganzach Y. Pazy A. (2014). Does core self evaluations predict career success? a reanalysis of Judge and Hurst (2008). *J. Res. Pers.* 48 107–115. 10.1016/j.jrp.2013.11.003

[B27] Gerhart B. A. Milkovich G. T. (1989). “Salaries, salary growth, and promotions of men and women in a large, private firm,” in *Pay Equity: Empirical Inquiries*, eds Michael R. T. Hartmann H. I. O’Farrell B. (Washington, DC: National Academy Press), 23–43.

[B28] Goldberg L. R. (1970). Man versus model of man: a rationale, plus some evidence, for a method of mproving on clinical inferences. *Psychol. Bull.* 73 422–432. 10.1037/h0029230

[B29] Gomez-Meijia L. R. Larraza-Kintatana M. Makei M. (2003). The deteminants of excecutive compensation in family controlled public corporations. *Acad. Manag. J.* 46 226–237. 10.5465/30040616 30040616

[B30] Grant A. M. Wrzesniewski A. (2010). I won’t let you down or Will I? core self-evaluations, other-orientation, anticipated guilt and gratitude, and job performance. *J. Appl. Psychol.* 95 108–121. 10.1037/a0017974 20085409

[B31] Greene C. N. (1973). Causal connections among managers’ merit pay, job atisfaction, and performance. *J. Appl. Psychol.* 58:95. 10.1037/h0035417

[B32] Hauser R. (1994). Measuring socioeconomic status in studies of child development. *Child Dev.* 65 1541–1545. 10.2307/1131279 7859541

[B33] Herrnstein R. J. Murray C. (1994). *The Bell Curve: Intelligence and Class Structure in American Life.* New York, NY: The Free Press.

[B34] Hinrichs J. R. (1969). Correlates of employee evaluations of pay increases. *J. Appl. Psychol.* 53 481–489. 10.1037/h0028655 5378216

[B35] Hogue M. DuBois C. L. Fox-Cardamone L. (2010). Gender differences in pay expectations: the roles of job intention and self-view. *Psychol. Women Q.* 34 215–227. 10.1111/j.1471-6402.2010.01563.x

[B36] Holliday J. Uher R. Landau S. Collier D. Treasure J. (2006). Personality pathology among individuals with a lifetime history of anorexia nervosa. *J. Pers. Disord.* 20 417–430. 10.1521/pedi.2006.20.4.417 16901263

[B37] Jaccard J. Helbig D. W. Wan C. K. Gutman M. A. Kritz-Silver-stein D. C. (1990). Individual differences in attitude-behavior consistency: the prediction of contraceptive behavior. *J. Appl. Soc. Psychol.* 20 575–617. 10.1111/j.1559-1816.1990.tb00428.x

[B38] Jonason P. K. Li N. P. Webster G. D. Schmitt D. P. (2009). The dark triad: facilitating a short-term mating strategy in men. *Eur. J. Pers.* 23 5–18. 10.1002/per.698

[B39] Judge T. A. Cable D. M. Boudreau J. W. Bretz R. D. (1995). An empirical investigation of the predictors of executive career success. *Pers. Psychol.* 48 485–520. 10.1111/j.1744-6570.1995.tb01767.x

[B40] Judge T. A. Erez A. Bono J. E. Thoresen C. J. (2003). The core self-evaluations scale (CSES): development of a measure. *Pers. Psychol.* 56 303–331. 10.1111/j.1744-6570.2003.tb00152.x

[B41] Judge T. A. Higgins C. Thoresen C. J. Barrick M. R. (1999). The big five personality traits, general mental ability, and career success across the life span. *Pers. Psychol.* 52 621–652. 10.1111/j.1744-6570.1999.tb00174.x

[B42] Judge T. A. Hurst C. (2007). Capitalizing on one’s advantages: role of core self-evaluations. *J. Appl. Psychol.* 92 1212–1227. 10.1037/0021-9010.92.5.1212 17845081

[B43] Judge T. A. Hurst C. (2008). How the rich (and happy) get richer (and happier): relationship of core self-evaluations to trajectories in attaining work success. *J. Appl. Psychol.* 93 849–863. 10.1037/0021-9010.93.4.849 18642988

[B44] Judge T. A. Hurst C. Simon L. N. (2009). Does it pay to be smart, attractive, or confident (or all three)?: relationships among general mental ability, physical attractiveness, core self-evaluations, and income. *J. Appl. Psychol.* 94 742–755. 10.1037/a0015497 19450010

[B45] Judge T. A. Kammeyer-Mueller J. D. (2011). Implications of core self-evaluations for a changing organizational context. *Hum. Resource Manag. Rev.* 21 331–341. 10.1016/j.hrmr.2010.10.003

[B46] Judge T. A. Klinger R. L. Simon L. S. (2010). Time is on my side: time, general mental ability, human capital, and extrinsic career success. *J. Appl. Psychol.* 95 92–107. 10.1037/a0017594 20085408

[B47] Judge T. A. Livingston B. A. (2008). Is the gap more than gender? A longitudinal analysis of gender, gender role orientation, and earnings. *J. Appl. Psychol.* 93 994–101. 10.1037/0021-9010.93.5.994 18808221

[B48] Kahneman D. Deaton A. (2010). High income improves evaluation of life but not emotional well-being. *Proc. Natl. Acad. Sci. U.S.A.* 107 16489–16493. 10.1073/pnas.1011492107 20823223 PMC2944762

[B49] Kuhberger A. Schulte-Mecklenbeck M. Perner J. (1999). The effects of framing, reflection, probability, and payoff on risk preference in choice tasks. *Organ. Behav. Hum. Decis. Process.* 78 204–231. 10.1006/obhd.1999.2830 10343064

[B50] Levy F. Murnane R. R. (1992). U.S. earnings levels and earnings inequality: a review of recent trends and proposed explanations. *J. Econ. Literat.* 30 1333–1381.

[B51] Loehlin J. C. Neiderhiser J. M. Reiss D. (2003). The behavior genetics of personality and the NEAD study. *J. Res. Pers.* 37 373–387. 10.1016/s0092-6566(03)00012-6

[B52] Lubinski D. Humphreys L. G. (1990). Assessing spurious “moderator effects”: Illustrated substantively with the hypothesized (”synergistic”) relation between spatial and mathematical ability. *Psychol. Bull.* 107 385–393. 10.1037/0033-2909.107.3.385 2349320

[B53] Luhmann M. Schimmack U. Eid M. (2011). Stability and variability in the relationship between subjective well-being and income. *J. Res. Pers.* 45 186–197. 10.1016/j.jrp.2011.01.004

[B54] Lykken D. T. (1968). Statistical significance in psychological research. *Psychol. Bull.* 70 51–59. 10.1037/h0026141 5681305

[B55] McClelland G. H. Judd C. M. (1993). Statistical difficulties of detecting interactions and moderator effects. *Psychol. Bull.* 114 376–390. 10.1037/0033-2909.114.2.376 8416037

[B56] Meehl P. (1959). A comparison of clinicians with five statistical methods of identifying psychotic MMPI profiles. *J. Counsel. Psychol.* 6 102–109. 10.1037/h0049190

[B57] Mitra A. Gupta N. Jenkins G. D. Jr. (1997). A drop in the bucket: when is a pay raise a pay raise? *J. Organ. Behav.* 18 117–137. 10.1002/(sici)1099-1379(199703)18:2<117::aid-job790>3.0.co;2-1

[B58] Mitra A. Tenhiälä A. Shaw J. D. (2016). Smallest meaningful pay increases: field test, constructive replication, and extension. *Hum. Resource Anagem.* 55 69–81. 10.1002/hrm.21712

[B59] Mosteller F. Tukey J. W. (1977). *Data Analysis and Regression.* Reading, MA: Addison Wesley.

[B60] Mulaik S. A. James L. R. Van Alstine J. Bennet N. Lind S. Stilwell C. D. (1989). Evaluation of goodness-of-fit indices for structural equations models. *Psychol. Bull.* 105 430–445. 10.1037/0033-2909.105.3.430

[B61] Parhhiban D. Kochhar R. Levitas D. (1998). The effect of institutional investors on the level and mix of CEO compensation. *Acad. Manag. J.* 41 200–208. 10.5465/257102 257102

[B62] Pearlin L. Lieberman M. Menaghan E. Mullan J. (1981). “Mastery scale,” in *Measures of Personality and Social Psychological Attitudes* (San Diego: Academic Press, Inc.).

[B63] Piliavin I. Gartner R. Thornton C. Matsueda R. (1986). Crime, deterrence, and rational choice. *Am. Sociol. Rev.* 51 101–119. 10.2307/2095480

[B64] Psacharopoulos G. Patrinos H. (2004). Returns to investment in education: a further update. *Educ. Econ.* 2 111–134. 10.1080/0964529042000239140

[B65] Resick C. J. Whitman D. S. Weingarden S. M. Hiller N. J. (2009). The bright-side and the dark-side of CEO personality: examining core self-evaluations, narcissism, transformational leadership, and strategic influence. *J. Appl. Psychol.* 94:1365. 10.1037/a0016238 19916649

[B66] Robinson M. D. Tamir M. (2005). Neuroticism as mental noise: a relation between neuroticism and reaction time standard deviations. *J. Pers. Soc. Psychol.* 89:107. 10.1037/0022-3514.89.1.107 16060749

[B67] Rosenberg M. (1961). The costs of acquisitiveness. *Psyccritiqes* 6 200–201. 10.1037/006619

[B68] Rosenberg M. (1965). Rosenberg self-esteem scale (RSE). Acceptance and commitment therapy. *Measur. Pack.* 61:18.

[B69] Rotter J. B. (1966). Generalized expectancies for internal versus external control of reinforcement. *Psychol. Monogr. Gen. Appl.* 80:1. 10.1037/h0092976 5340840

[B70] Russell C. J. Dean M. A. (2000). To log or not to log: bootstrap as an alternative to the parametric estimation of moderation effects in the presence of skewed dependent variables. *Organ. Res. Methods* 3 166–185. 10.1177/109442810032002

[B71] Seibert S. E. Crant J. M. Kraimer M. L. (1999). Proactive personality and career success. *J. Appl. Psychol.* 84 416–427. 10380421 10.1037/0021-9010.84.3.416

[B72] Seibert S. E. Kraimer M. L. Liden R. (2001). A social capital theory of career success. *Acad. Manag. J.* 44 219–237. 10.2307/3069452

[B73] Spurk D. Abele A. E. (2011). Who earns more and why? A multiple mediation model from personality to salary. *J. Bus. Psychol.* 26 87–103. 10.1007/s10869-010-9184-3

[B74] Stevens S. S. (1946). On the theory of scales of measurement. *Science* 103 677–680. 10.1126/science.103.2684.677 17750512

[B75] Stevens S. S. (1958). Problems and methods of psychophysics. *Psychol. Bull.* 55 177–196. 10.1037/h0044251 13567963

[B76] Stroh L. K. Brett J. M. Baumann J. P. Reilly A. H. (1996). Agency theory and variable pay compensation strategies. *Acad. Manag. J.* 39 751–767. 10.5465/256663

[B77] Stumpp T. Muck P. M. Hülsheger U. R. Judge T. A. Maier G. W. (2010). Core self-evaluations in Germany: validation of a German measure and its relationships with career success. *Appl. Psychol.* 59 674–700. 10.1111/j.1464-0597.2010.00422.x

[B78] Sutin A. R. Costa P. T. Miech R. Eaton W. W. (2009). Personality and career success: concurrent and longitudinal relations. *Eur. J. Pers.* 23 71–84. 10.1002/per.704 19774106 PMC2747784

[B79] Thurstone L. L. (1929). Fechner’s law and the method of equal appearing intervals. *J. Exper. Psychol.* 12 214–222. 10.1037/h0070968

[B80] Trostel I. Walker I. Woolley P. (2002). Estimates of the economic return to schooling for 28 countries. *Lab. Econ.* 1 580–593.

[B81] Tsang E. W. K. Kwan K. M. (1999). Replication and theory development in organizational science: a critical realist perspective. *Acad. Manag. Rev.* 24 759–780. 10.2307/259353

[B82] Turban D. B. Greening D. W. (1996). Corporate social performance and organizational attractiveness to prospective employees. *Acad. Manag. J.* 40 658–672. 10.2307/257057

[B83] Van Dyck C. Frese M. Baer M. Sonnentag S. (2005). Organizational error management culture and its impact on performance: a two-study replication. *J. Appl. Psychol.* 90 1228–1240. 10.1037/0021-9010.90.6.1228 16316276

[B84] Wilk S. Burris D. Sackett P. (1995). Gravitation to jobs commensurate with ability: longitudinal and cross-sectional tests. *J. Appl. Psychol.* 80 79–85. 10.1037/0021-9010.80.1.79

[B85] Wilk S. Sackett P. R. (1996). Longitudinal analysis of ability-job complexity fit and job change. *Pers. Psychol.* 49 837–967.

[B86] Worley C. G. Bowen D. E. Lawler E. E. III (1992). On the relationship between objective increases in pay and employees’ subjective reactions. *J. Organ. Behav.* 13 559–571. 10.1002/job.4030130603

[B87] Zedeck S. Smith P. C. (1968). A psychophysical determination of equitable payment: a methodological study. *J. Appl. Psychol.* 52:343. 10.1037/h0026245 5681111

[B88] Zhang A. Arvey R. D. (2009). Effects of personality on individual earnings: leadership role occupancy as a mediator. *J. Bus. Psychol.* 24 271–280. 10.1007/s10869-009-9105-5

